# Estimating willingness to pay for public health insurance while accounting for protest responses: A further step towards universal health coverage in Tunisia?

**DOI:** 10.1002/hpm.3505

**Published:** 2022-05-23

**Authors:** Mohammad Abu‐Zaineh, Olivier Chanel, Khaled Makhloufi

**Affiliations:** ^1^ Aix Marseille Univ, CNRS, AMSE Marseille France; ^2^ Faculty of Medical and Paramedical Sciences, Aix‐Marseille Univ. Marseille France; ^3^ Doha Institute for Graduate Studies Doha Qatar; ^4^ Aix‐Marseille University, Faculty of Medicine – Timone, INSERM‐IRD‐UMR 912 (SESSTIM) Marseille France

**Keywords:** contingent valuation, protest answers, self‐selection, universal health coverage, willingness‐to‐pay

## Abstract

**Introduction:**

Developing countries face major challenges in implementing universal health coverage (UHC): a widespread informal sector, general discontent with rising economic insecurity and inequality and the rollback of state and public welfare. Under such conditions, estimating the demand for a health insurance scheme (HIS) on voluntary basis can be of interest to accelerate the progress of UHC‐oriented reforms. However, a major challenge that needs to be addressed in such context is related to protest attitudes that may reflect, *inter alia*, a null valuation of the expected utility or unexpressed demand.

**Methods:**

We propose to tackle this by applying a contingent valuation survey to a non‐healthcare‐covered Tunisian sample *vis‐à‐vis* joining and paying for a formal HIS. Our design pays particular attention to identifying the nature of the willingness‐to‐pay (WTP) values obtained, distinguishing genuine null values from protest values. To correct for potential selection issues arising from protest answers, we estimate an ordered‐Probit‐selection model and compare it with the standard Tobit and Heckman sample selection models.

**Results:**

Our results support the presence of self‐selection and, by predicting protesters' WTP, allow the “true” sample mean WTP to be computed. This appears to be about 14% higher than the elicited mean WTP.

**Conclusion:**

The WTP of the poorest non‐covered respondents represents about one and a half times the current contributions of the poorest formal sector enrolees, suggesting that voluntary participation in the formal HIS is feasible.

## Highlights

- We study how protest positions may impact affiliation to formal health insurance
- We apply a contingent valuation survey to a non‐healthcare‐covered population
- We account and correct for selection issues with appropriate econometric models
- Accounting for protest positions is found to impact the implementation of UHC

## 1 INTRODUCTION

On 21 May 2013, the World Bank Group President Dr. Jim Yong Kim set the goal for universal health coverage (UHC): to “*close the gap in access to health services and public health protection for the poorest 40% of the population in every country*”.^1^ Any plan to expand health coverage in the Middle East and North Africa (MENA) region will obviously need to address the key issue of the health financing policies. Alami^2^ showed that reforms seeking to extend current health coverage on a contributory and formal employment basis are unlikely to succeed, leaving swathes of the active working population without any health coverage. As in other countries in the MENA region, *de jure* entitlement to the Tunisian National Health Insurance Fund “*Caisse Nationale d’Assurance Maladie – CNAM*” – is mainly based on formal sector employment.^2^, ^3^ Remarkable efforts have been made to extend health insurance coverage, through the medical assistance plan, which is means‐tested benefit, and the formal‐mandatory health insurance regime of *CNAM* for both employed and self‐employed. However, the uptake in the informal sector, which represents about 40% of the labour force and 50% of the young population,^4^, ^5^ remains severely limited by the *ad hoc* selection criteria and restrictions on entitlements (i.e., the prerequisite condition of declared activity).^6^ The continuing large‐scale non‐coverage of the informal sector may also be due to a protest attitude towards the continuing prevalence of high out‐of‐pocket payments, the rise of private healthcare providers due to the rollback of state and public welfare, and the lack of confidence in the government's capacity to deliver the healthcare needed.^7^, ^8^, ^9^, ^10^, ^11^ Yet the field lacks a systematic analysis of how protest positions may impact health financing policies based on voluntary participation, and hence, the implementation of UHC‐oriented reforms.

We propose to fill this gap by using a Contingent Valuation (CV) survey to elicit preferences from the non‐covered Tunisian population regarding a voluntary health insurance scheme (VHIS) (see Nosratnejad et al.^12^ for a review of willingness‐to‐pay (WTP) for health insurance in low‐ and middle‐income countries (LMICs)). Stated preference methods help inform decision‐makers on the hypothetical demand for not yet available or non‐market health products or services. Analyses, however, generally focus on valid WTP, frequently paying insufficient attention to the impact of “protest” WTP (with few exceptions^13^, ^14^). The latter may result from the respondent's protest beliefs or attitudes *vis‐à‐vis* the survey or the proposed scenario,^15^, ^16^, ^17^ the payment vehicle *per se*,^18^ or, for UHC, the lack of faith in institutions^19^, ^20^ and in the capacity of governments to ensure the provision of good healthcare for all.^21^ These protest answers differ from the stated “genuine null WTP” – indicating the respondent's unwillingness to join due to either a budget constraint or a null valuation of the expected utility – because they hide an unexpressed demand. We use an ordered‐Probit‐selection model (OPS) to properly disentangle genuine null values from protest answers and estimate the true demand for a VHIS by incorporating predictions for protest WTP.^22^

We find evidence of self‐selection issues reflecting protest behaviours against some components of the survey (e.g., the subject, the principle of the interview, the methodology …), or because of lack of confidence in the government or the health insurance provider. Our results suggest that the segments of the population not covered by any health insurance scheme (HIS) would be willing to join the current formal scheme. In particular, using the OPS model, we find that the WTP of the poorest non‐covered population represents about one and a half times the formal sector's poorest enrolees' contributions. Accounting for the often‐unexpressed demand of the non‐covered population may thus help expand the breadth of coverage of the formal HIS on a voluntary basis, and hence accelerate the progress towards achieving UHC in Tunisia,^23^ and more broadly in the LMICs.

## 2 METHODS

A CV study was conducted in Tunisia between August 1^st^ and 30 September 2013, with the aim of eliciting the willingness‐to‐join (WTJ), and to‐pay for, on a voluntary basis, the current formal HIS run by CNAM. The inclusion criterion comprises any Tunisian citizens not covered by a HIS. Most of the non‐covered segments of the Tunisian population belong to the informal sector, defined as the proportion of work not covered by any form of social security (i.e., pension and/or health insurance).^11^, ^24^ Informal workers represent about 40% of the Tunisian active workforce (almost 1.1million), most of them are young under 40 years old (60% of males and 83% of females). There is an effective rate of 56.7% social insurance coverage in the private sector (79.1% of employees and 39.3% of self‐employed).^5^

**FIGURE 1:**
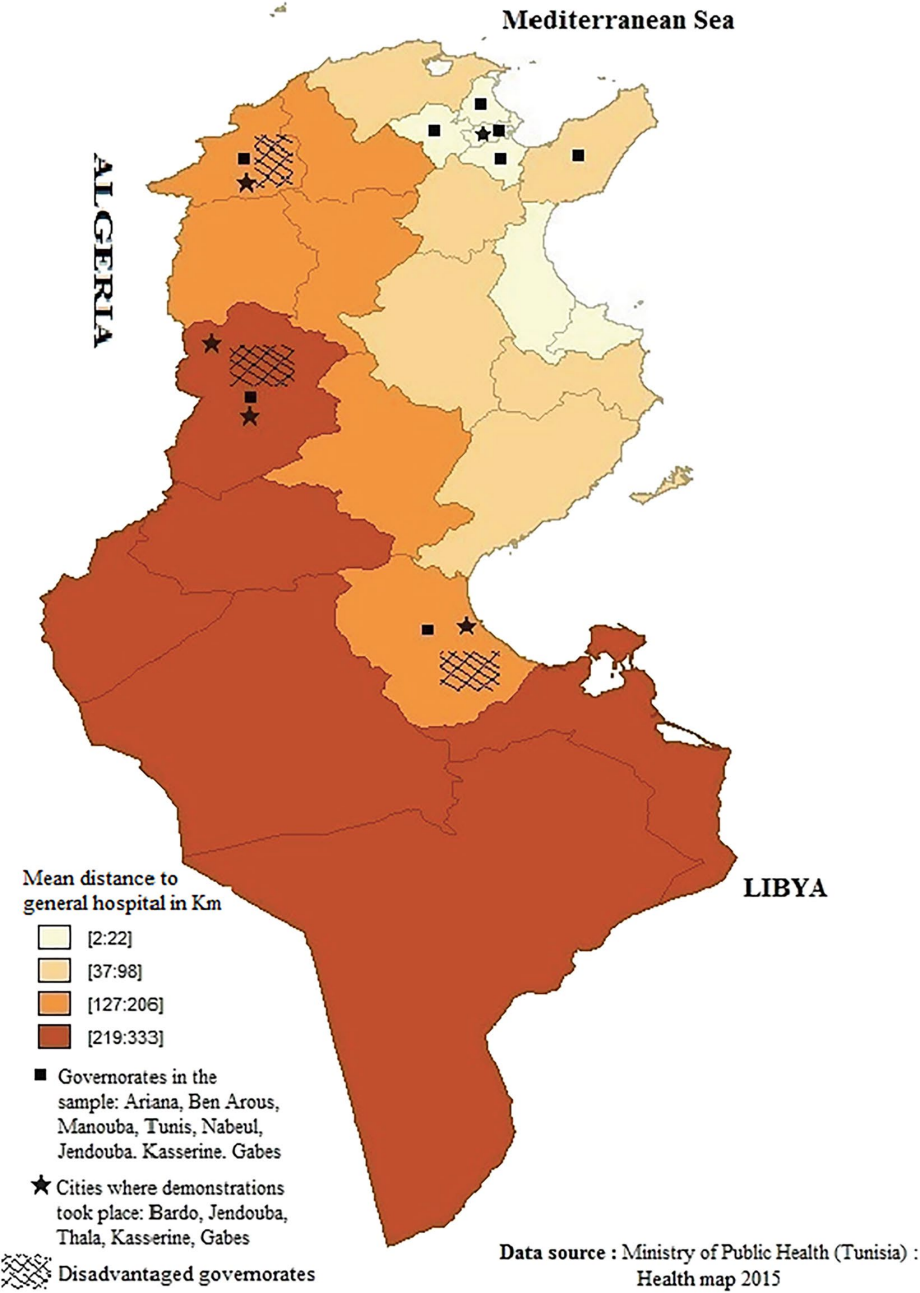
Map of Tunisia showing governorates in the sample

The sample covered 456 respondents, of whom 30 refused to participate, resulting in a response rate of 93.42%. Given the targeted population, 385 was the minimum sample size required for a 5% margin of error and a 95% confidence interval. Because this population can be hard‐to‐reach, we implemented the venue‐based method of time‐space sampling technique.^25^ It entails selecting places and times where respondents gather the most. Accordingly, the CV has been conducted in two primary places: (1) ‘*Souks*’ – the traditional marketplaces in which informal workers are concentrated; (2) ‘*Al‐Mydan’* – the public squares where unemployed people are likely to gather and demonstrate. At the time of the study, none of the respondents were covered by any of the available HIS. By randomly selecting participants from these two meeting places in the three regions of Tunisia (North, Central and South), the implemented sampling technique thus ensured the representativity of the main non‐covered groups of the population (i.e., informal sector's workers and unemployed). Additionally, in order to take into account the observed differences in access to health care facilities across Tunisia,^23^, ^26^ eight governorates were sampled over the three regions of Tunisia (see Figure 1).

The survey instrument was developed and refined using two pre‐tests (pilots). The questionnaire was administered face‐to‐face by well‐trained interviewers. After presenting the main objectives of the CV study, respondents were asked to give the reason(s) why they are not covered by the current national HIS administrated by the CNAM. Following the public single‐provider scheme, the CNAM proposes coverage for healthcare facilities, with a cap imposed on the annual health expenditure amount.^3^ Respondents were then asked to reveal their preferences *vis‐à‐vis* voluntary enrolment in an already existing HIS run by the CNAM. If respondents were not willing to join, they were then asked to state the reason(s) and whether they would join the scheme if it was offered for free or at a very low cost. This particular design was intended to help distinguish between genuine null WTP values and protest answers and to avoid falsely classifying the latter as null WTP responses. Non‐protesters were asked to state their maximum WTP values.

Three different elicitation techniques were employed to gather information on respondents' WTP values: the well‐known Open‐Ended (OE) and Payment Card (PC) techniques,^27^, ^28^ and a variant known as the Circular Payment Card (CPC).^29^, ^30^ The CPC uses a visual representation of a circular card with no pre‐determined start or end points. Accordingly, the interviewer presents the CPC at a random position, and asks respondents to spin it until they find the bracket that best corresponds to their WTP values. The rationale for employing these three elicitation techniques stems from the fact that the information collected on the respondents' WTP tend to be affected by the format of these techniques. Comparisons between the results obtained from these three elicitation techniques have been carefully examined in two companion papers.^29^, ^30^ Finally, the questionnaire gathered information on the respondents' socio‐economic, socio‐demographic and health characteristics. In addition, respondents were asked to state their opinions on the proposed insurance scheme. All respondents were asked to give their full consent to participate in the study and no financial incentives were offered.

The overall sample was randomly split into three equal and mutually exclusive sub‐groups to respond to the above‐mentioned three WTP elicitation formats. The range and centring of the bids on the PC and CPC were chosen based on the WTP values already elicited through two pre‐tests. The appropriate strategy for modelling WTP values hinges on the specific format chosen to elicit these values.^31^, ^32^ While the OE format involves stating a point estimate of WTP, the PC and CPC state an interval with two specified thresholds. Thus, the actual elicited WTP value can be used in the case of OE, while with PC and CPC this value can be reliably approximated using the middle of the bid‐range elicited.^33^, ^34^, ^35^

The WTP stated by respondent i, $WTP_{i}$, corresponds to the amount that would equalise her/his *ex‐ante* utility level (i.e., before joining the VHIS) and her/his *ex‐post* utility level (i.e., after joining the VHIS), also known as the Hicksian compensating measure. $WTP_{i}$ can be either positive, null, or missing. Some null values may simply reflect genuine values, indicating the respondent's unwillingness‐to‐join the scheme due to either a budget constraint or a null valuation of her/his expected utility change. In this case, when the question is whether s/he would join a scheme that is either offered for free or at a low cost, answering with a ‘Yes’ is expected (hence, a genuine null WTP). By contrast, missing and some WTP values may rather conceal some protest attitude on the part of the respondent *vis‐à‐vis* at least one of the following: the interview, the policy intervention, the methodology/characteristics of the survey (e.g., the proposed scheme, the public provider, the benefit package), or a strategic behaviour, *viz*., free‐riding.^16^, ^17^, ^36^ In this case, the respondent alludes to her/his unwillingness‐to‐participate by refusing to join even at a null or a very low cost (hence, a protest answer). These intrinsically different significances reflect two different behaviours. First, a rational behaviour with a zero demand for VHIS. Second, a self‐selection issue that may conceal a potential non‐zero (unexpressed) demand. Consequently, these two types of responses should be accounted for and distinguished in any econometric strategy to adequately estimate the demand for VHIS. In what follows, we present econometric models that properly account for these two types (see Supplementary Appendix for a detailed presentation).

We first consider, as a benchmark, the Tobit model on non‐protest answers, which distinguishes positive WTP from genuine zero WTP.^37^ However, given that respondents with non‐protest WTP may not be randomly drawn from the whole sample of respondents, we need to account for the possible correlation between variables explaining non‐protestation and the unobserved individuals' heterogeneity, that is, sample selection issues.

We then consider the standard Heckit sample selection model that simultaneously specifies a Probit‐type equation for selection (predicting whether or not the respondent is willing to join the VHIS for free or at very low cost, WTJ) and a linear‐type equation for WTP conditional on the individual not protesting.^38^

Finally, the OPS model allows continuous variables to be observed based on an ordered selection rule. It was derived from Terza's^39^ seminal work, and mainly used since then to account for selection based on performance indicators.^22^, ^40^, ^41^ The OPS model can however also account for selection induced by protest answers in CV surveys.^42^, ^43^ The first equation (selection) models the probability of giving non‐protest answers with an ordered Probit, distinguishing between genuine null WTP (those who are willing to join for free or at very low cost), protest answers with unobserved WTP, and strictly positive WTP values. The second equation (outcome) is linear, conditional on strictly positive WTP.

The estimations present no particular difficulties when the log‐likelihood is maximised using standard unconstrained optimization methods.^44^

## 3 RESULTS

Table 1 provides descriptive statistics on respondents' socio‐economic and demographic characteristics as well as their needs and preferences *vis‐à‐vis* the proposed HIS. Interestingly, while 34% of the respondents declare that they do not have any insurance coverage due to the complexity of administrative procedures, about 49% state that their lack of insurance coverage is due to the fact that their professional activities are not officially declared. Lastly, it is worth noting that about 44% of the respondents live in disadvantaged governorates (see also Figure 1).

**TABLE 1:** Descriptive statistics (*n* = 426)

Variable definition	Mean (Std. dev.)
**Dependent variables**	
WTP for VHIS (quarterly, in TND) if WTP>0 (*n* = 336)	42.58 (25.34)
WTP for VHIS (quarterly, in TND) if WTP≥0 (*n* = 394)	36.32 (27.85)
**Respondent characteristics**	
Male = **1** if male, **0** if female	0.669 (0.471)
Age = individual's age (in years)	35.384 (10.394)
Household size = number of household members	2.598 (2.011)
Child = **1** if at least one child under 5 years old in the household, **0** otherwise	0.133 (0.340)
Elderly = **1** if there is a person more than 65 years old in the household, **0** otherwise	0.051 (0.221)
Married = **1** if Married, **0** otherwise	0.417 (0.493)
NoSchool = **1** No schooling, **0** otherwise	0.023 (0.151)
Elementary = **1** primary school, **0** otherwise	0.213 (0.410)
Secondary = **1** secondary education, **0** otherwise	0.516 (0.500)
High school = **1** higher education, **0** otherwise	0.246 (0.431)
Household income = Monthly household income (in TND)	558.11 (464.15)
Individual income = Monthly respondent income (in TND)	539.14 (456.87)
Equivalized Income^a^ = [Monthly income/(household size)^0.5^] (in TND)	425.80 (425.72)
Work = **1** if employed/self‐employed, **0** otherwise	0.788 (0.408)
Rural = **1** living in rural area, **0** otherwise	0.197 (0.398)
DisadGov.^b^ = **1** living in disadvantaged governorate, **0** otherwise	0.443 (0.497)
**Other variables**	
NonDeclared = **1** uninsured due to no declared work, **0** otherwise	0.490 (0.500)
Administration = **1** uninsured due to administrative procedures, **0** otherwise	0.340 (0.474)
NoNeed = **1** uninsured due to no need, **0** otherwise	0.663 (0.198)
RiskAverse = **1** if risk‐averse, 0 otherwise^c^	0.885 (0.319)
**Respondent‐specific health variables**	
Self‐reported health status = **1** if self‐reported health status is good, **0** otherwise	0.835 (0.371)
Outpatient respondent = **1** if at least one outpatient care during the last 3 months, **0** otherwise	0.380 (0.486)
Inpatient respondent = **1** if at least one hospitalisation during the last 8 months, **0** otherwise	0.093 (0.292)
Chronic condition = **1** if respondent reports a chronic condition,**0** otherwise	0.124 (0.330)
FinancialHealth = **1** if can afford health services, **0** otherwise	0.370 (0.483)
Smoking = **1** if consuming tobacco products, **0** otherwise	0.460 (0.498)
**Health variables specific to the family members of the resp.**	
Outpatient member = 1 if at least one outpatient care in household during the last 3 months, 0 otherwise	0.5 (0.500)
Inpatient member = 1 if at least one hospitalisation in household during the last 8 months, 0 otherwise	0.140 (0.348)
Chronic member = 1 if one household member reports a chronic condition, 0 otherwise	0.185 (0.389)
**Survey specific variables**	
OE** = 1** if open‐ended elicitation format, **0** otherwise	0.331 (0.471)
PC** = 1** if payment card elicitation format, **0** otherwise	0.322 (0.468)
CPC** = 1** if circular payment card elicitation format, **0** otherwise	0.347 (0.477)
PublicSquare = **1** if sample point is a public square, **0** if informal market	0.420 (0.494)
Interviewer#1–5 = Dummy variables for each of the 5 interviewers	‐

Abbreviations: CPC, Circular Payment Card; OE, Open Ended; PC, Payment Card; TND, Tunisian Dinar; WTP, Willingness To Pay.

^a^ Equivalized income is computed based on the OECD equivalence scale, by dividing household income by the square root of household size.^45^

^b^ According to decree *n*° 2008‐387 of 11 February 2008, see Figure 1.

^c^ If among the three most risk averse modalities out of six generated.^46^

About 58 respondents (13.5%) reveal a willingness‐to‐join the proposed VHIS if it was offered for free or at very low cost but give null WTP (genuine null WTP). About 7.5% (32 respondents) show a protest attitude, refusing to join the proposed VHIS even for free (protest WTP). The quarterly mean WTP of the non‐protest respondents is 36.32 TND (at the time of the survey, 1 TND (Tunisian Dinar) = € 0.455 = $ 0.605), and 42.58 TND when computed on strictly positive WTP values. Finally, the median WTP (including the genuine null WTP) is 35 TND, which means that, on average, 50% of the respondents are willing‐to‐pay about 2.16% (that is, ((35TND×4)/(539×12)) per quarter of their income to join the VHIS.

In the following, we start from full models with all explanatory variables: survey‐specific variables (interviewer effect, survey point and elicitation format), respondents' socio‐economic, socio‐demographic and health characteristics (health status, use of healthcare and ability to pay for treatment for a given illness, risk aversion and reasons for not being covered by health insurance). Only five interviewers covered the eight sample locations (see Figure 1), generating strongly imbalanced distributions of the *Rural*, Disadvantaged governorate (*DisadGov*) and Sample point (*PublicSquare*) variables by interviewer, with two consequences. First, to avoid high collinearity, we do not use these three spatially related variables in the same model. Second, the interviewer effect likely reflects a particular combination of these variables in addition to a potential interviewer effect. Parsimonious models are obtained after removing explanatory variables step by step by decreasing *p*‐values. All significant variables in the full model remain significant in the parsimonious models (details upon request).

In the Tobit model estimated over the sub‐sample of non‐protest WTP (see Table A in Supplementary Appendix), as expected, household equivalent income appears to be significantly correlated with WTP. This provides evidence of the validity of the stated preference survey.^47^ Similarly, WTP values appear to be significantly positively associated with individuals' employment status (*Work*), risk aversion attitude (*RiskAverse*), ability to afford health services (*FinancialHealth)* in addition to the presence of one chronic condition in the family (*Chronic member)*. Unsurprisingly, individuals declaring no need for health insurance (*NoNeed)* appear to be less willing to pay for VHIS. Finally, a statistically significantly negative effect is also found for the two survey‐specific variables, with interviewer 2 and OE and PC elicitation formats leading to lower WTP values compared to other interviewers and CPC format, respectively.^30^

A Heckman model is estimated over the whole sample and explicitly accounts for the potential effect of protest answers (see Table A in Supplementary Appendix). It is worth noting, first, that the two equations predicting the WTJ and the WTP are significantly negatively correlated (*p*‐value = 0.029). While this gives support to the self‐selection hypothesis, it indicates that the selection‐specific variables negatively affect the WTP through the correlation parameter ρ. WTJ the VHIS appears to be significantly negatively associated with household equivalent income, living in a rural area, and declaring no need for insurance. By contrast, both education level and living in a disadvantaged governorate are positively associated with WTJ the VHIS. As regards the survey‐specific variables, WTJ is affected positively by interviewer 3 and negatively by the PC elicitation format.

For those willing to join the VHIS, we find a significantly positive effect of household equivalent income, employment status, and the presence of a chronic condition on WTP. WTP values tend, however, to decrease with smoking status and living in a rural area, and also with some survey‐specific variables (Interviewer 2 and OE and PC formats).

Results in Table 2 are obtained from estimating the OPS model over the whole sample. The OPS model independently accounts for the issues related to both genuine null WTP and protest answers. Average marginal effects (AME) are given for each of the three modalities of the ordered Probit: Pr(genuinenullWTP), Pr(ProtestWTP) and Pr(WTP>0). Overall, results show similar patterns to those obtained from the Heckit and the Tobit models. In particular, the presence of a chronic condition significantly increases Pr(WTP>0) and decreases Pr(genuinenullWTP) and Pr(ProtestWTP), whereas living in a rural area and declaring no need for health insurance have the opposite effects. A new variable enters the selection equation‐ having at least one child under 5 years old at home –and is negatively associated with Pr(genuinenullWTP) and Pr(ProtestWTP) but positively associated with Pr(WTP>0) and WTP amount. Also of note, the correlation coefficient between the selection and WTP equations does not significantly differ from zero. Regarding the marginal effects on WTP, we found the same results (significance and sign) as in the Heckit and Tobit models.

**TABLE 2:** Regression results: Ordered Probit selection model

	Model 2					
	Selection equation: Ordered probit				WTP equation	
Variables	Parameter (*p*‐value)	AME on Pr(WTP = 0)	AME on Pr (protest)	AME on Pr(WTP>0)	Parameter (*p*‐value)	AME on WTP
NoNeed (=1)	−1.271***	0.215***	0.070***	−0.285***		−2.817***
	(<0.0001)	(<0.0001)	(<0.0001)	(<0.0001)		(<0.0001)
Chronic member (=1)	0.495**	−0.084**	−0.027**	0.111**		1.096**
	(0.033)	(0.034)	(0.038)	(0.032)		(0.028)
Child (=1)	0.777***	−0.132***	−0.043***	0.174***		1.723***
	(0.006)	(0.006)	(0.009)	(0.005)		(0.004)
Rural (=1)	−0.594***	0.101***	0.033***	−0.133***		−1.317***
	(0.002)	(0.002)	(0.003)	(0.002)		(0.003)
Interviewer 2 (=1)	−1.111***	0.188***	0.061***	−0.249***		−19.776***
	(<0.0001)	(<0.0001)	(<0.0001)	(<0.0001)		(<0.0001)
Cut off 1	−1.744***					
	(<0.0001)					
Cut off 2	−1.373***					
	(<0.0001)					
Constant					22.937***	
					(<0.0001)	
OE format (=1)					−5.844**	−5.844**
					(0.035)	(0.035)
PC format (=1)					−6.892**	−6.892**
					(0.014)	(0.014)
Equiv. Revenue (euros)					0.0231***	0.0231***
					(<0.0001)	(<0.0001)
Work (=1)					9.618***	9.618***
					(0.001)	(0.001)
RiskAverse (=1)					9.636***	9.636***
					(0.008)	(0.008)
FinancialHealth (=1)					5.592**	5.592**
					(0.027)	(0.027)
Rho (ρ)					−0.156	‐
					(0.422)	
No. obs.	426					
Joint nullity test (*p*‐value)	227.45*** (<0.0001)					
lnL_0_						
Log likelihood	−1726.824					

Abbreviations: AME, average marginal effects; OE, Open Ended; PC, Payment Card; WTP, Willingness To Pay.

* if *p* < 0.10, ** if *p* < 0.05, *** if *p* < 0.01.

One advantage of using the OPS model estimates is the ability to compute the adjusted‐WTP values that account for both genuine null WTP and selection issues in relation to protest WTP values. Table 3 shows that the predicted WTP for protesters is on average TND 51.82, leading to a sample mean WTP of TND 38.2 once null and protest answers are accounted for, 14% more than the observed TND 33.59. When expressed as a fraction of 2013 Tunisian gross national income per capita,^48^ our sample showed an average WTP of 2.13%, in the range 0.35%–6.07% found over 11 LMICs.^12^, ^49^ Finally, it is worth noting that the mean predicted WTP for protesters is higher than for non‐protesters.

**TABLE 3:** Observed and predicted WTP (in TND) based on Ordered Probit selection model

	Mean protest WTP (*n* = 32)	Genuine null WTP (*n* = 58)	Mean positive WTP (*n* = 336)	Mean WTP with null WTP (*n* = 394)	Mean WTP with null and protest (*n* = 426)
Observed WTP	− (−)	0 (0)	42.58 (25.34)	36.32 (27.85)	33.59^a^ (28.44)
Predicted WTP	51.82 (18.42)	0^b^ (0)	43.49 (14.13)	37.09 (20.21)	38.20 (20.43)

Abbreviations: TND, Tunisian Dinar; WTP, Willingness To Pay.

Standard deviation in brackets.

^a^ Wrongly assuming that protest WTPs are equal to 0.

^b^ Setting predicted values to 0.

## 4 DISCUSSION

This paper attempted to unravel the societal, true demand for HIS by appropriately accounting for null and missing WTP values that may conceal unexpressed demand underlying the respondents' protest attitude. This is especially relevant in countries like Tunisia with a large informal sector (about 40% of the workforce) and high unemployment (about 15.3% of the workforce in 2013).^50^, ^51^ Furthermore, the country has witnessed during the last decade several protests and social unrests reflecting a general discontent with the overall state of the public welfare and the economy.

Our CV survey proposed an HIS coverage to respondents who did not express urgent healthcare needs; thus, the obtained WTP is likely to be closer to their true ability‐to‐pay.^52^, ^53^ Indeed, we find that WTP values tend to increase with individuals' income, level of education and poor health status, in accordance with a review on WTP for HIS in Asian and Sub‐Saharan African countries.^12^ Our results also shed light on the importance of protest WTP, even though the 7.5% in our sample were somewhat less than the 13.3% found in a study on WTP for community‐based health insurance among informal workers in urban Bangladesh.^54^ Accounting for these protest answers can help reveal the true demand for a VHIS, hence the implementation of UHC‐oriented reforms and the health financing policies.

We compare existing HIS contributions at the time of the survey – whose rates differ depending on the individual's income and employment status – and our predicted WTP for the proposed VHIS. Based on the official CNAM rate for the third quarter of 2013, the quarterly SHI contribution for “low‐income independent” workers with quarterly income below the reference TND 580.4 (affiliated under the special regime implemented in 2002), is TND 14.51. In our sample, the 34 respondents in this category are willing‐to‐pay about one and a half times this formal sector enrolee's contribution: TND 24.11 (standard deviation, SD 14.25). Regarding “independent workers” with quarterly income above the reference TND 580.4, they pay a quarterly HIS contribution of TND 58.76. The 392 respondents in this category are willing to pay more than low‐income respondents – TND 39.42 (SD 20. 34) versus TND 24.11 – although less than the existing contribution of TND 58.76. This shows that a voluntary participation in a formal HIS may be acceptable to the majority of the uninsured subpopulations. However, the continuing reliance on payroll contributions from formal employment has long hindered efforts to extend coverage to certain subpopulations such as informal workers, the self‐employed, the unemployed and the vulnerable.

Moreover, it is not only the breadth of coverage that matters, but also the depth of coverage and the explicit entitlement to a specific package of services that can fulfil the population's needs.^55^ Protest attitudes here seem to be driven by non‐income factors: the lack of adequate quality health care facilities in rural areas, as well as the view of some respondents that they do not need health insurance. Our results corroborate existing evidence that enrolment in HIS does not solely depend on income, but on other factors such as the availability of healthcare services and their spatial distributions across the different regions of the country.^2^, ^56^, ^57^ Indeed, such finding echoes the protestors' general discontent as regards the unequal distribution of healthcare facilities: high‐level public‐sector healthcare providers are mostly concentrated in urban centres where the better‐off segments of the population benefit the most.

Although the path towards UHC is context‐specific, lessons drawn from our results can be of interest for other LMICs seeking to attain UHC. Efforts to promote UHC need to be accompanied by a parallel improvement in the spatial distribution of healthcare services. The priority should be changing the modality of enrolment and the depth of coverage. Indeed, it is increasingly argued that UHC may not be achieved based on contributory enrolment alone. Allowing enrolment on a voluntary basis while accounting for protest attitudes and subsidizing the enrolment of some groups of the population can accelerate progress towards UHC. However, the issue of the optimal financing‐mix of UHC remains open for future research.^10^, ^58^, ^59^ It is worth noting that the financing source of the various subsidized health insurance arrangements can come from other sources in addition to central government revenues: regional/provincial/district public authorities, social assistance allowances, charitable organisations, or donor funds (through government budget support programs, and civil society).

## CONFLICT OF INTEREST

The authors declare that they have no conflict of interest.

## ETHICS STATEMENT

No ethical approval was required at the time of the survey, because the dataset is anonymous and handled in accordance with the “Database and Privacy” law, Number 78–17 of 6 January 1978, concerning data processing and privacy. In addition, the formal consent to participate has been obtained from each respondent and data were used in an aggregate way.

## Supporting information

Supplementary Material 1

## Data Availability

Research data are not shared.
