# Supplementary material for: Estimating willingness to pay for public health insurance while accounting for protest responses: A further step towards universal health coverage in Tunisia?
Source: Int J Health Plann Manage. 2022 May 23;37(5):2809–21. doi: 10.1002/hpm.3505 (PMC9542895; doi:10.1002/hpm.3505)
Supplement: Supplementary file 1 — Supplementary Material 1 [file HPM-37-2809-s001.docx]

**Supplementary Material Statistical Appendix**

**Estimating willingness to pay for public health insurance while accounting for protest responses: A further step towards universal health coverage in Tunisia?**

The Tobit model that distinguishes positive WTP from genuine zero WTP modeled the WTP as a latent variable WTP_i_* as follows:

${WTP}_{i}^{*}=X_{i}^{'}\beta+\varepsilon_{i}$; $\varepsilon_{i}\sim N(0,\sigma_{\varepsilon}^{2})$ (1)

where X_i_ is a set of explanatory variables specific to respondent *i*, ε_i_ is the error term, ${WTP}_{i}={WTP}_{i}^{*}$ if ${WTP}_{i}^{*}>0$, and ${WTP}_{i}=0$, otherwise. The expected $WTP$ is given by

$${E[WTP}_{i}\left| X_{i} \right]=\Phi\left( {x_{i}^{'}\beta}/\sigma\right)X_{i}^{'}\beta+\sigma\phi({x_{i}^{'}\beta}/\sigma)$$

where $\phi$ is the density function of the standard normal distribution and $\Phi$ is the corresponding cumulative distribution function. The pair (${WTP}_{i}$, $X_{i}$) is observed when respondent $i$ chooses to join the VHIS.

The sample selection model simultaneously specifies a probit-type equation for selection (willing to join the VHIS for free or at low cost, ${WTJ}_{i}$) and a linear-type equation for WTP conditional on the individual not being a protester as follows (Heckman, 1976):

${WTJ}_{i}^{*}=Z_{i}^{'}\alpha+u_{i}$; $u_{i}\sim N(0,\sigma_{u}^{2})$ (2)

where ${WTJ}_{i}$=1 if $WTJ_{i}^{*}>0$ and ${WTJ}_{i}$= 0 if $WTJ_{i}^{*}\leq0.$ $Z_{i}$ is a set of variables explaining the decision of individual $i$ to join the VHIS and u_i_ is the error term.

The $WTP$ given ${WTJ}_{i}$=1 can then be estimated as:

${WTP}_{i}=X_{i}^{'}\beta+\varepsilon_{i}$; $\varepsilon_{i}\sim N(0,\sigma_{\varepsilon}^{2})$ (3)

Equation (3) is estimated on the subsample of non-protest WTP. The joint distribution of error terms in equations (2) and (3) is assumed to be:

$$(u, \varepsilon)\sim N[(0, 0, 1, \sigma_{\varepsilon}^{2}, \rho)$$

where $\sigma_{u}^{2}$ is normalized to 1, and $\rho$is the correlation coefficient between the two error terms. The expected $WTP$ accounting for selection is given as:

${E[WTP}_{i}\left| X_{i}, Z_{i}, {WTJ}_{i}=1 \right]=X_{i}^{'}\beta+\rho\sigma_{\varepsilon}\lambda(Z_{i}^{'}\alpha)$ (4)

where $\lambda\left( Z_{i}^{'}\alpha\right)=\phi({Z_{i}^{'}\alpha)}/{\Phi\left( Z_{i}^{'}\alpha\right)}$ is the inverse Mills ratio. If the estimated correlation coefficient, $\hat{\rho}$, is not statistically significantly different from zero, then the two error terms are independent. This suggests that selection bias resulting from the protest attitudes is not a problem; hence other modeling strategies estimating equations (2) and (3) separately (e.g., two-part model) may be relevant (Madden, 2008).

The ordered-probit-selection (OPS) distinguishes between genuine null WTP (those who are willing to join for free or at low cost) and protest answers with unobserved WTP. Consider the underlying latent model:

${y_{i}^{*}=W}_{i}^{'}\alpha+u_{i}$ (5)

where $y_{i}$is observed as per the following ordered-probit selection rule,

$$y_{i}=\left\{ \begin{aligned} 0 \left( genuine null WTP \right) if c_{0}\leq y_{i}^{*}<c_{1} \\ 1 \left( protest WTP \right) if c_{1}\leq y_{i}^{*}<c_{2} \\ 2 \left( WTP>0 \right) if c_{2}\leq y_{i}^{*}<c_{3} \end{aligned} \right.$$

where $c_{0}\equiv-\infty$ , $c_{3}\equiv\infty$and $c_{1}$ and $c_{2}$ are estimated simultaneously along with parameter $\alpha$. The ${WTP}_{i}$ is not observed if $y_{i}=0, 1,$while strictly positive *WTP_i_* values (if y_i_ = 2) can be modeled using equation (3). The error terms of the ordered-probit and the OLS equations are assumed to follow a bivariate normal distribution $(u, \varepsilon)\sim N[(0, 0, 1, \sigma_{\varepsilon}^{2}, \rho)$, where the variance of $u$ is normalized to 1 to allow identification; $\rho,$is the linear correlation coefficient between $\varepsilon$ and $u$ and $\sigma_{\varepsilon}^{2}$ is the variance of $\varepsilon$. The expected $WTP$ conditional on an observed strictly positive WTP is given as:

${E[WTP}_{i}\left| X_{i}, W_{i}, y_{i}=2 \right]=X_{i}^{'}\beta+\left\{ {[1-\Phi(c_{2}-W_{i}^{'}\alpha-\rho\sigma_{\varepsilon})]}/{[1-\Phi(c_{2}-W_{i}^{'}\alpha)}] \right\}$ ( 6 )

**References**

Heckman, J. (1976). The Common structure of statistical models of truncation, sample selection and limited dependent variables and a simple estimator for such models. *Annals of Economic and Social Measurement*, 5(4), 120–137.

Madden, D. (2008). Sample selection versus two-part models revisited: the case of female smoking and drinking. *Journal of Health Economics,* 27(2), 300-307.

**Table A: Regression results (Tobit and Heckman selection models)**

|  | **Tobit Model** | | **Heckman selection model** | | | |
| --- | --- | --- | --- | --- | --- | --- |
|  |  |  | Selection equation (Probit) | | WTP equation | |
| **Variables** | Parameter  (*p*-value) | Marginal effect | Parameter  (*p*-value) | Marginal effect | Parameter  (*p*-value) | Marginal Effect |
| Constant | 22.144*** | - | 1.268*** | - | 32.121*** | - |
|  | (<0.0001) | - | (<0.0001) | - | (<0.0001) | - |
| OE format (=1) | -6.904** | -5.931** | - | - | -5.129* | -5.129* |
|  | (0.039) | (0.039) | - | - | (0.085) | (0.085) |
| PC format (=1) | -7.769** | -6.673** | -0.541** | -0.051** | -7.265** | -7.840*** |
|  | (0.015) | (0.015) | (0.023) | (0.017) | (0.011) | (0.005) |
| Equiv. income (TND) | .01784*** | .01533*** | -0.00047* | -0.000044* | 0.01874*** | .01824*** |
|  | (<0.0001) | (<0.0001) | (0.055) | (0.055) | (<0.0001) | (<0.0001) |
| Work (=1) | 10.574*** | 9.083*** | - | - | 10.648*** | 10.648*** |
|  | (0.001) | (0.001) | - | - | (<0.0001) | (<0.0001) |
| FinancialHealth (=1) | 7.237** | 6.216** | - | - | - | - |
|  | (0.025) | (0.023) | - | - | - | - |
| Tobacco (=1) | - | - | - | - | -5.189** | -5.189** |
|  |  |  | - | - | (0.029) | (0.029) |
| NoNeed (=1) | -17.780*** | -15.273*** | -1.113*** | -.106*** | - | -1.181* |
|  | (0.003) | (0.003 | (<0.0001) | (<0.0001) | - | (0.072) |
| Chronic member (=1) | 6.365* | 5.467* | - | - | 6.682** | 6.682** |
|  | (0.070) | (0.070) | - | - | (0.040) | (0.040) |
| Elementary(=1) | - | - | 1.000** | 0.095** | - | 1.061** |
|  |  | - | (0.029) | (0.018) | - | (0.036) |
| Secondary(=1) | - | - | .834*** | .079*** | - | 0.885** |
|  |  | - | (0.005) | (0.001) | - | (0.030) |
| Rural(=1) | - | - | -0.630** | -0.052** | -7.578** | -8.246*** |
|  |  |  | (0.019) | (0.012) | (0.013) | (0.007) |
| DisadGov. (=1) | - |  | 0.978*** | 0.093*** | - | 1.038** |
|  |  |  | (<0.0001) | (<0.0001) | - | (0.049) |
| DumGamb (=1) | 8.037* | 6.904* | - | - | - | - |
|  | (0.063) | (0.061) |  | - |  | - |
| Interviewer 2 (=1) | -28.407*** | -24.401*** | - | - | -21.822*** | -21.822*** |
|  | (<0.0001) | (<0.0001) | - | - | (<0.0001) | (<0.0001) |
| Interviewer 3 (=1) | - | - | 1.086** | 0.103** | - | 1.152 |
|  |  | - | (0.022) | (0.011) | - | (0.125) |
| Sigma (σ) for Tobit | 26.084 | - | - | - | -0.276** | - |
| (Rho (ρ) for Heckman |  | - | - | - | (0.029) | - |
| No. obs. | 394 |  | 426 | | | |
| Joint nullity test  (p-value) | 18.03***  (<0.0001) |  | 160.14***  (<0.0001) | | | |
| lnL_0_ | - |  | -1885.846 | | | |
| Log likelihood | -1629.94 |  | -1876.262 | | | |

OE: Open Ended. PC: Payment Card. * if p<0.10, ** if p<0.05, *** if p<0.01.
